# The Intelligibility of Interrupted Speech: Cochlear Implant Users and Normal Hearing Listeners

**DOI:** 10.1007/s10162-016-0565-9

**Published:** 2016-04-18

**Authors:** Pranesh Bhargava, Etienne Gaudrain, Deniz Başkent

**Affiliations:** 1 Department of Otorhinolaryngology/Head and Neck Surgery, University of Groningen, University Medical Center Groningen, P.O. Box 30.001, 9700 RB Groningen, The Netherlands; 2 Graduate School of Medical Sciences, Research School of Behavioral and Cognitive Neurosciences, University of Groningen, P.O. Box 30.001, 9700 RB Groningen, The Netherlands; 3 Lyon Neuroscience Research Center, CNRS UMR 5292, Inserm U1028, Auditory Cognition and Psychoacoustics, Université Lyon 1, 50 av. Tony Garnier, 69366 Lyon Cedex 7, France

**Keywords:** interrupted speech, cochlear implants, speech perception, cochlear implant simulations, aging, glimpsing

## Abstract

Compared with normal-hearing listeners, cochlear implant (CI) users display a loss of intelligibility of speech interrupted by silence or noise, possibly due to reduced ability to integrate and restore speech glimpses across silence or noise intervals. The present study was conducted to establish the extent of the deficit typical CI users have in understanding interrupted high-context sentences as a function of a range of interruption rates (1.5 to 24 Hz) and duty cycles (50 and 75 %). Further, factors such as reduced signal quality of CI signal transmission and advanced age, as well as potentially lower speech intelligibility of CI users even in the lack of interruption manipulation, were explored by presenting young, as well as age-matched, normal-hearing (NH) listeners with full-spectrum and vocoded speech (eight-channel and speech intelligibility baseline performance matched). While the actual CI users had more difficulties in understanding interrupted speech and taking advantage of faster interruption rates and increased duty cycle than the eight-channel noise-band vocoded listeners, their performance was similar to the matched noise-band vocoded listeners. These results suggest that while loss of spectro-temporal resolution indeed plays an important role in reduced intelligibility of interrupted speech, these factors alone cannot entirely explain the deficit. Other factors associated with real CIs, such as aging or failure in transmission of essential speech cues, seem to additionally contribute to poor intelligibility of interrupted speech.

## **INTRODUCTION**

In everyday listening scenarios, when the target speech is masked by fluctuating background noise or competing speech, normal-hearing (NH) listeners are able to employ top-down mechanisms to perceptually restore the masked speech information. This phenomenon, variously referred to as ‘glimpsing’, ‘dip-listening’ or ‘listening-in-the-valleys’, implies that the listeners are able to track and integrate the glimpses of unmasked target speech portions into a speech stream using the spectro-temporal cues and additional linguistic and contextual information from the unmasked segments (Miller and Licklider 1950; Buus 1985; Moore 2003; Srinivasan and Wang 2005; Wang and Humes 2010; Başkent and Chatterjee 2010; Başkent 2012). Thus, the intelligibility of speech in noisy listening scenarios depends on listeners’ ability to integrate the speech samples from successive glimpses.

Noisy scenarios pose challenging listening conditions to cochlear implant (CI) users in understanding speech. CI users have limited spectro-temporal resolution, often accompanied by other distortions arising due to electrode placement, degree of neural survival and front-end signal processing features (Qin and Oxenham 2003; Nelson and Jin 2004; Başkent and Shannon 2005; Başkent and Shannon 2006). Due to this, the spectro-temporal cues in the intact portions of speech are degraded for CI users (Rosen 1989; Fu and Shannon 2000; Friesen et al. 2001). In noisy scenarios, reduced intelligibility of intact portions and degraded bottom-up cues may inhibit the tracking and integration of the glimpses of unmasked target speech portions into a speech stream. This could cause a difficulty in employing the top-down mechanisms in restoring and understanding degraded speech.

A failure in integrating and restoring the speech stream from glimpses may help explain, at least partially, the difficulty CI users experience in understanding speech in a noisy scenario. Keeping this in view, the current study was designed to test integration and restoration of glimpses from interrupted speech in CI users in comparison with NH listeners. In speech, glimpsing and restoration have been studied using masking paradigms in which a speech stream is periodically masked by temporally fluctuating noise or competing talker’s speech stream (see Assmann and Summerfield, 2004, for a review) or, alternatively, using phonemic restoration (Bhargava et al. 2014). The fluctuating masker affects CI users more than NH listeners (Nelson et al. 2003; Nelson and Jin 2004; Fu and Nogaki 2005). This could be due to the failure of CI users to deal with obliterated speech itself, i.e. failure in tracking and integrating the glimpses (Nelson and Jin 2004; Gnansia et al. 2010) and/or due to various additional deleterious effects of the masker, e.g. the spectral smearing that occurs in electrical stimulation, making the target speech blend more in the masker (Fu et al. 1998; Friesen et al. 2001; Fu and Nogaki 2005), and weaker transmission of voice cues that can be otherwise helpful in segregating target speech from the masker (Stickney et al. 2007; Fuller et al. 2014).

One way to test glimpsing without additional deleterious effects of a masker is using speech periodically interrupted with silent intervals (Nelson and Jin 2004). Even though no noise is involved in interrupted-speech perception, the listener has to employ top-down mechanisms to integrate and restore unavailable speech portions. These are likely the same mechanisms involved in masking paradigm and real-world noisy scenario. Jin and Nelson (2010) found a strong correlation between the scores of sentence recognition in noise and interrupted sentence recognition for both NH listeners and hearing-impaired listeners. This similarity makes interrupted-speech perception paradigm a good technique to learn more about common mechanisms listeners use in understanding speech in a noisy scenario (Iyer et al. 2007; Jin and Nelson 2010; Wang and Humes 2010), without the potentially harmful effects of added noise on speech perception (Nelson and Jin 2004). Despite these advantages, interrupted-speech perception is less frequently studied (Miller and Licklider 1950; Shafiro et al. 2011) and even lesser so with CI users in conjunction with sentences (Nelson and Jin 2004; Wang and Humes 2010). For these reasons, in the present study, we used interrupted-speech perception paradigm to test integration and restoration of glimpses in CI users and do a comparison with NH listeners.

In NH listeners, depending on the speech material, speaker rate and on-cycle duration, speech intelligibility shows a U shape. In general, many words remain intact at slow interruption rates (<2 Hz), while multiple looks per words are available through interruptions at fast rates (>4 Hz). These two conditions thus result in good intelligibility. At intermediate rates (2–4 Hz), almost every or every other word is obliterated, causing a drop in intelligibility (Powers and Speaks 1973; Huggins 1975; Nelson and Jin 2004; Shafiro et al. 2011). Miller and Licklider (1950) and Wang and Humes (2010) reported that the intelligibility of single words was lowest at the rate of around 4 Hz because entire phonemes were eliminated at this rate. Faster interruption rate resulted in better intelligibility. Similarly, Powers and Wilcox (1977) found that the intelligibility of sentences was highest when the interruption rate was such that the listener received at least one partial look at every word.

In contrast to NH, for CI users, there have been fewer studies on perception of interrupted speech. Evidence from the limited literature indicates that silent interruptions strongly disrupt the intelligibility of speech for CI users. For instance, Chatterjee et al. (2010) used 5-Hz interruption rate with sentences, and Gnansia et al. (2010) used 4-Hz interruption rate with nonsense bisyllables to find that CI listeners’ intelligibility of interrupted speech was drastically lower than that of NH listeners. This indicates that, compared with NH listeners, CI users experience more difficulty in integrating spectro-temporal cues across silent interruptions at word as well as segmental level. A possible explanation for this was proposed by Gilbert et al. (2007) who found that for speech with envelope cues only, the lowest intelligibility is for the slow and medium interruption rates of 2–4 Hz, suggesting the involvement of modulation masking with envelope-only cues. Since CI users rely heavily on envelope cues, slow and medium rates of interruption are likely to produce modulation masking disrupting intelligibility for CI users than NH listeners. Thus, a wide range of rates of interruption has to be used to also adequately explore the effect of silent interruptions in CI users while allowing for the possibility of observing the effects of modulation masking.

Nelson and Jin (2004) used a range of slow and fast interruption rates (between 1 and 32 Hz), with sentences, to compare CI users’ intelligibility of interrupted speech with NH listeners presented with and without noise-band vocoded speech. At interruption rates faster than 4 Hz, the scores for NH listeners listening to full-spectrum speech were significantly high, while the scores for CI users and four-channel noise-band vocoded speech listeners were close to the floor level. At interruption rates slower than 4 Hz, the intelligibility scores of NH listeners listening to full-spectrum speech dropped significantly, while the scores of CI users and NH listeners listening to vocoded speech remained close to the floor level. Because noise-band vocoding may have overestimated the detrimental effect of spectro-temporal degradation of speech stimulus (Shamma and Lorenzi 2013), it is likely that NH listening to vocoded speech had performed worse than CI users. Because of the floor effect, it was not clear in their study if the silent interruptions affected the noise-band vocoded speech listeners and CI users similarly.

There were a couple of factors not analysed by Nelson and Jin that could also have contributed to the findings, namely, potential effects of aging and lower baseline perception of (uninterrupted) speech in CI users. Although recent work has failed to demonstrate a detrimental effect of aging on speech intelligibility in steady or fluctuating noise maskers for NH listeners (Schoof and Rosen 2014), aging is shown to be accompanied with decline in auditory temporal processing (Fitzgibbons and Gordon-Salant 1996; Saija et al. 2014). In light of the reliance on temporal envelope cues by CI listeners and the competing decline in temporal auditory perception in aging adults, aging may be deemed as a highly relevant factor for speech perception in CI users. Given that the participants involved in the study by Nelson and Jin had a considerable age difference between the NH group (19–32 years) and the CI group (34-64 years, mean age 49 years), differing performances may have been partially due to aging. Secondly, in the study by Nelson and Jin, no significant correlation was found between the intelligibility of uninterrupted speech and that of interrupted speech for the CI users. However, since the performance of CI users with interrupted speech was at floor level, this correlation was also difficult to interpret. In addition to this, the baseline intelligibility with uninterrupted speech for CI users was lower than the baseline intelligibility for NH listeners presented with four-channel noise-band vocoded speech. As a result, based on the existing literature, it is still unclear whether only the loss of spectro-temporal resolution simulated with vocoding (and not other factors such as aging, disparity in speech intelligibility baseline, etc.) could account for the performance of CI users.

One more factor that is known to affect interrupted speech intelligibility in NH listeners is the duty cycle of speech signal. Varying the duty cycle manipulates the availability of additional speech cues, and, this way, one can measure how well the listeners can utilize this additional information. Longer duty cycle provides longer intact speech portions containing more spectro-temporal cues as compared to shorter duty cycle. In NH listeners, longer duty cycle has been reported to lead to better integration of glimpses and restoration of obliterated speech portions (Miller and Licklider 1950; Wang and Humes 2010; Shafiro et al. 2011). In CI users, longer duty cycle (75 %) has been reported to provide not only better intelligibility of interrupted speech as compared to shorter duty cycle (50 %) but also significant restoration of speech with filler noise (Bhargava et al. 2014). Since these studies show that listeners can make use of longer duration speech cues for intelligibility, especially for CI users, duty cycle should be considered an important aspect of interrupted-speech perception. No previous study involving CI users explored duty cycle with a wide range of interruption rates, and, as a result, it is not clear whether CI users would be able to consistently take advantage of better duty cycle for the perception of interrupted speech at various interruption rates.

Thus, there is evidence that CI users have reduced interrupted-speech perception as compared to NH listeners, but the factors that may influence this reduction are not yet fully understood. In this study, we have systematically investigated perception of interrupted speech by CI users for a range of interruption rates and duty cycle, while also taking into account the effects of other potential factors.

We divided the study into three parts, each dealing with one research question. The first research question is “How does the interrupted-speech perception of CI users differ from the interrupted-speech perception of NH listeners?” In this regard, in experiment 1, we aimed to systematically characterize the extent of potential deficits in perception of periodically interrupted speech that the CI users have as compared to the NH listeners presented with normal speech (NHnorm). By using a range of interruption rates, and different duty cycles of the interruptions, we obtained a comprehensive picture of how the CI users deal with the temporal interruptions, given that their access to speech is already degraded due to the limitations of electrical stimulation. The second research question is “Can the deficit in interrupted-speech perception be explained on the basis of low spectro-temporal resolution availed through CI devices?” To answer this, in experiment 2, a control group of young NH listeners was tested with eight-channel noise-band vocoded simulations of CI processing (NHVoc), and their performance was compared with that of CI users. Through this, we explored whether the deficit in understanding interrupted speech was merely a result of the reduced spectro-temporal resolution, which would be indicated by similar performances between the two groups. In case a difference in their performance was found, there would be a possibility of a combined effect with other factors related to the actual CIs, such as aging, channel interaction, front-end processing, the quality of signal transmission at the electrode-nerve interface and residual hearing. Related to this, the third research question was “Could the disparity in interrupted-speech perception between CI users and NH listeners be contingent on more factors than only the loss of spectro-temporal details?” To answer this, in experiment 3, we aimed to test the factors of aging and baseline intelligibility (as a combined effect of other CI-related factors listed above). This time, a new control group of NH listeners (NHVocM) was tested with the noise-band vocoding; however, these were matched in age to the experiment group and, further, their baseline performance with uninterrupted speech was also matched to individual CI users by using noise-band vocoding with individualized spectral resolution and filter order. The data for CI users was collected once and then used for comparisons with NHnorm (experiment 1), NHVoc (experiment 2) and NHVocM (experiment 3).

## **EXPERIMENT 1. PERCEPTION OF INTERRUPTED SPEECH COMPARED BETWEEN CIs AND NORMAL-HEARING (NHnorm)**

The first experiment was run in order to fully capture potential deficits CI users may have in perceiving interrupted speech, as characterized over a range of combination of interruption rates and duty cycles. These were compared to the control data from young NH listeners presented with normal full-spectrum speech (NHnorm), i.e. only interrupted, but with no noise-band vocoding.

### Participants

Eight CI users (six males and two females; 28 to 75 years; average age 53.8) comprised the CI group. They were recruited via the clinic of the Otorhinolaryngology Department, University Medical Center Groningen. The demographics of these participants are shown in Table 1. All CI participants were monaurally implanted and had more than 1 year of CI experience with their device prior to the experiment. They were selected to represent typical CI users. The only exception was the inclusion criterion of relatively high speech perception performance so that the effects of interruption rate and duty cycle could be fully observed with minimal floor effect. For this purpose, CI users with phoneme-identification score above 65 % were recruited (Table 2). Phoneme-identification scores were measured in the clinic using *Nederlandse Vereniging voor Audiologie* (NVA) word corpus (Bosman 1989). The word corpus consists of lists of words, each containing 12 monosyllabic Dutch words of the pattern Consonant-Vowel-Consonant, spoken by a female talker. Each speech sound correctly identified is scored. The first word of each list serves as the introductory word and is excluded from scoring. Some of our participants had acoustic hearing at the lowest audiometric frequencies of 250 and 500 Hz (Table 2). Three CI users had hearing aids prescribed for their nonimplanted ear, but these were not used during data collection.

**TABLE 1:** Details of CI participants

Subject ID	Gender	Age during experiment (years)	Age at onset of hearing loss (years)	Whether prescribed hearing aid in nonimplanted ear	Duration of CI usage (years)	CI brand (and processor) AB = advanced bionics
CI1	M	53	0	No	2	AB HiRes 90K Helix (Harmony)
CI2	M	71	46	Yes	2	AB HiRes 90K Helix (Harmony)
CI3	M	33	1	Yes	8	Cochlear CI24R CS (Freedom)
CI4	F	75	53	No	7	Cochlear CI24R CA (Freedom)
CI5	M	61	37	No	4	Cochlear CI24RE CA (Freedom)
CI6	M	71	68	No	3	Cochlear CI24RE CA (Freedom)
CI7	F	28	3	No	10	Cochlear CI24R CS (Esprit3G)
CI8	M	38	3	Yes	1.5	Cochlear CI24RE CA

‘n.a.’ indicates that the item was not available in the CI user's clinical file or that the CI user could not provide the information

**TABLE 2:** Pre-operative hearing thresholds of the nonimplanted ear for CI participants vis-à-vis their phoneme-identification scores and sentence-identification baseline scores

Subject ID	Pre-operative tone thresholds of nonimplanted ear (dB HL)						Clinical phoneme-identification scores At 75 dB SPL (%)	Experimental sentence-identification baseline scores At 60 dB(A) (%)
	250 Hz	500 Hz	1000 Hz	2000 Hz	4000 Hz	8000 Hz		
CI1	75	85	85	115	120	*	67	70
CI2	n.a.	85	85	75	70	95	80	86
CI3	80	100	110	115	115	*	95	99
CI4	30	50	90	90	95	*	91	98
CI5	*	115	95	90	90	105	85	99
CI6	60	60	50	100	*	*	n.a.	97
CI7	95	105	110	120	*	*	75	83
CI8	70	85	110	115	130	110	85	93.8

An asterisk (*) denotes where the threshold was not measurable because of very poor hearing, while ‘n.a.’ denotes that the readings were not available

Eight young NH listeners (five males and three females; 19 to 28 years; average age 22.4) comprised the control group. The NH participants had an average pure-tone hearing threshold (across the test frequencies of 0.5, 1, 2 and 4 kHz) at the better ear lower than or equal to 20 dB HL. They were recruited through a database of participants who had participated in similar behavioural experiments at our lab but had never been exposed to the specific test materials or procedures of the present study.

All participants were native speakers of Dutch with no language disorders.

### Stimuli

The stimuli were grammatically well-formed, meaningful and highly contextual Dutch sentence recordings from the sentence corpus prepared by Versfeld et al. (2000). The sentences were spoken by a male talker and recorded digitally at 44.1 kHz. The speaker rate was on average about three words per second. Thirty-nine lists, each comprising 13 sentences, were provided with the corpus. The first three lists of the sentence corpus were always used for training the participants and for measuring the sentence-identification baseline score with uninterrupted sentences. For the main experiments, 12 lists per subject were randomly chosen from the remaining 36 lists.

### Signal Processing

**FIG. 1:**
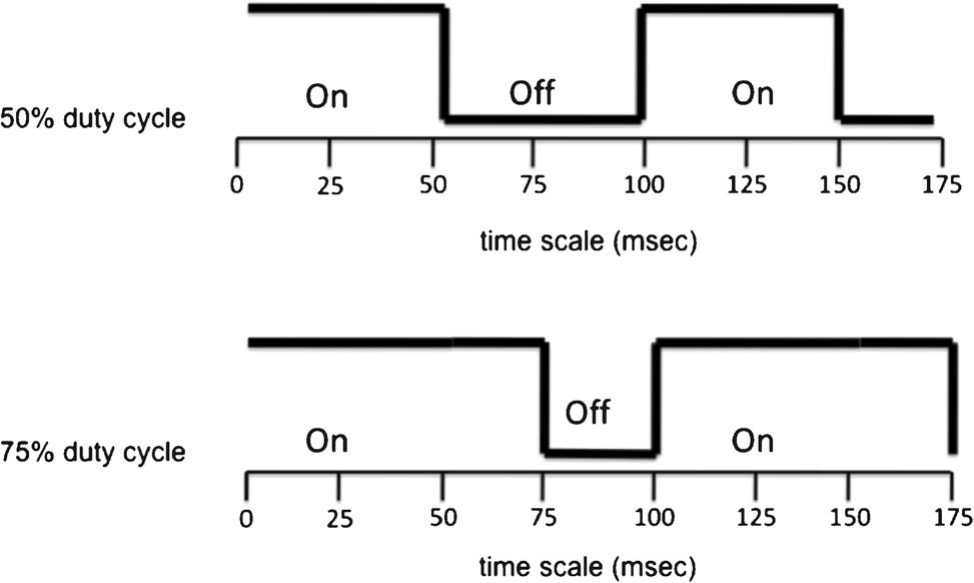
A schematic diagram of gating function used in the study, shown for 10 Hz interruption. The *top panel* shows the square-wave gating function with the 50 % duty cycle, i.e. with 50 % on-duration and 50 % off-duration. The *lower panel* shows the same with the 75 % duty cycle, i.e. with 75 % on-duration and 25 % off-duration.

The speech stimuli were periodically interrupted with silent gaps in a manner similar to our previous studies (Başkent and Chatterjee 2010; Bhargava and Başkent 2012). The interruptions were produced by modulating the sentence recordings with periodic square waves (ramping time of 5 ms) that varied on two parameters: rate of interruption (1.5, 3, 6, 10, 12 and 24 Hz) and duty cycle (50 and 75 %, representing the “on” time relative to the period; see Fig 1). The range of the interruption rates was chosen to produce both poor (slow rates) and good (fast rates) intelligibility for the NH listeners, based on a pilot study and past studies (Miller and Licklider 1950; Powers and Speaks 1973; Nelson and Jin 2004; Gilbert et al. 2007; Başkent 2010; Chatterjee et al. 2010; Başkent and Chatterjee 2010).

### Experimental Setup

The participants sat in an anechoic chamber, approximately 1 m away from the loudspeaker, facing a computer monitor and the loudspeaker. The stimuli were routed via the S/PDIF output of an external soundcard, Echo AudioFire 4 (Echo Digital Audio Corporation, California, USA), and presented through a single active Tannoy Precision 6D (digital) loudspeaker (Tannoy Ltd, UK) in free field, at 60 dB (A) (measured with a KEMAR head and torso at the position where the participant is seated).

### Procedure

The general procedure of the experiment was similar to Başkent and Chatterjee (2010). The sentences were processed and presented using MATLAB. The participants listened to one stimulus sentence at a time and verbally repeated what they heard. They were encouraged to guess in their responses when not certain. The spoken responses were recorded digitally and scored off-line. The percent correct scores were calculated as the ratio of the number of correctly identified words to the number of total words per list. There was no penalty for no or incorrect identification of the words. The raw percent correct scores were then converted into rationalized-arcsine units (RAU; Studebaker (1985)). Given the average number of words for the sentence lists, the maximum possible RAU score was 118 and the minimum possible RAU score was −18.

The experiment comprised 12 test conditions (six interruption rates × two duty cycles). For each condition, one list of 13 sentences was used, resulting in 156 sentences for the 12 conditions. A short alert tone preceded every stimulus. To provide a preview of the test condition to the listener, the same introductory sentence, processed in the same way as that list, preceded every list of sentences. This introductory sentence was not included in the calculation of intelligibility scores. The order of the conditions was randomized for each participant. The sentence-identification baseline was measured with two lists of sentences, without silent intervals. The entire session was completed within 2 h by each participant. For familiarization with the procedure of the experiment, a short training with different signal-processing parameters than the main experiment (0.75-Hz interruption rate at 40 % duty cycle) was provided. One list of sentences, which was the same for all participants, was used for training. Feedback was not provided during the training or during data collection.

The CI users were tested with their regular clinical device. During the training session, they adjusted their device to the setting they found most comfortable. This setting was then not changed during the main experiment.

### Statistical Analyses

Statistical analyses were performed on the transformed RAU scores. For measuring the significance of the main effects and the interactions, ANOVAs were run on SPSS (IBM Corp., Release 18.0.0), as it allowed for applying Greenhouse-Geisser correction. Post hoc false-discovery-rate (FDR)-corrected two-tailed *t* tests were run for multiple comparisons on R software package (R Foundation for Statistical Computing, Release 2.15.1). To compare the experimental interruption conditions with the baseline condition with uninterrupted sentences, Dunnett’s test was used, also run on R software.

### Results and Discussion

**FIG. 2:**
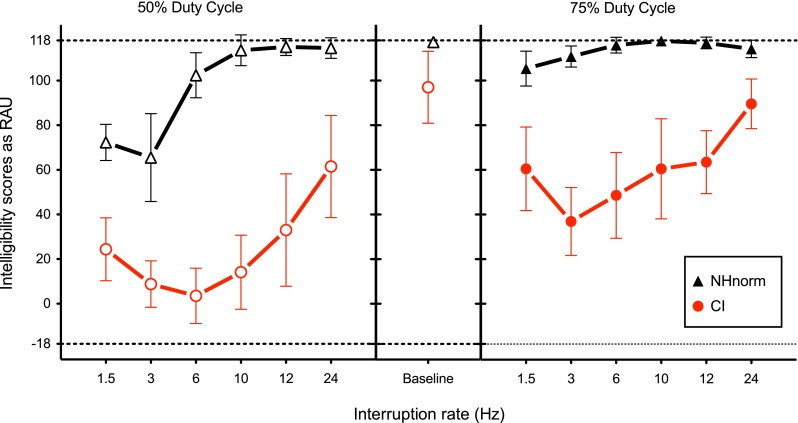
The mean interrupted-sentence intelligibility scores in RAU plotted for NHnorm (*black triangle*) and CI (*red circle*) as a function of interruption rates. The results for the two duty cycles are shown in the *left* (50 %) and *right* (75 %) panels. Sentence-identification baseline scores for uninterrupted sentences are shown in the *middle panel*. The *error bars* denote one standard deviation.

Figure 2 shows the data for the CI and the NHnorm groups as a function of interruption rates and separately for the 50 and the 75 % duty cycles in the left and right panels. The sentence-identification baseline scores with no interruptions are shown in the middle panel.

The data from NHnorm listeners shows that, similar to previous studies (Miller and Licklider 1950; Nelson and Jin 2004; Bhargava and Başkent 2012), the intelligibility of interrupted speech remained nearly as high as the uninterrupted sentence-identification baseline score, except for a small number of interruption conditions. Confirming this, Dunnett’s test showed that for 50 % duty cycle, except for slow interruption rates, the performance was not significantly different from the baseline condition [for all comparisons <10 Hz, *p* < 0.05; 10 Hz, *t*_*d*_ = 0.74, *p* = 0.94; 12 Hz, *t*_*d*_ = 0.44, *p* = 0.99; 24 Hz, *t*_*d*_ = 0.53, *p* = 0.98]. Similar results were found for 75 % duty cycle as well [6 Hz, *t*_*d*_ = 0.61, *p* = 0.97; 10 Hz, *t*_*d*_ = 0.3, *p* = 0.99; 12 Hz, *t*_*d*_ = 0.14, *p* = 0.991; 24 Hz, *t*_*d*_ = 1.49, *p* = 0.48; all other comparisons, *p* < 0.05].

Overall, the performance of CI participants was lower than that of NH participants. The average sentence-identification baseline score for the CI group was significantly lower than that for NH listeners tested with full-spectrum speech [CI and NHnorm; *t*_7.17_ = 3.51, *p*_adj_ = 0.025]. Interrupting the speech with silent intervals further deteriorated the intelligibility for the CI users for both the duty cycles. Dunnett’s test showed that all interruption conditions produced a significantly different performance than the baseline condition with uninterrupted speech [*p* < 0.0001], except for 75 % at 24 Hz [*t*_d_ = 1.62, *p* = 0.39].

The intelligibility scores were the highest for the faster rates of interruption (24 Hz) and lowest for medium rates of interruption (3 and 6 Hz), indicating that CI users were able to make use of more glimpses per second. The intelligibility scores were higher for slowest interruption rate of 1.5 Hz as compared with the medium rates. This nonmonotonicity was not observed with NH listeners with 75 % duty cycle, due to ceiling effect. A paired sample *t* test [*t*_5_ = −10.52, *p* < 0.05] found the intelligibility with 75 % duty cycle [mean = 59.92] to be significantly higher than the intelligibility with 50 % duty cycle [mean = 24.22].

Thus, for several conditions tested in this study, the CI users were not only able to successfully integrate and restore the speech segments interrupted by silent intervals but were also able to achieve better intelligibility of interrupted speech by taking advantage of the faster and longer sampling of speech signal. To compare the adverse effect of silent interruptions between the groups of CI users and NH listeners tested with full-spectrum speech, a two-way mixed model ANOVA was used to analyse the data for each duty cycle, with interruption rate as within-subject factor and mode of hearing (i.e. NHnorm vs. CI) as between-subject factor (Table 3). For both the duty cycles, a significant main effect of interruption and mode of hearing, and a significant interaction were found. Thus, the CI users’ intelligibility of interrupted speech was significantly lower than that of the NHnorm listeners, and the interruption rate affected the intelligibility for CI listeners differently than NHnorm listeners, for both of the duty cycles. To make sure that these results were not caused only by the ceiling effects, the three slowest interruption rates at 50 % duty cycle—where there was no saturation for either group—were re-examined. This analysis showed that the interaction between interruption rate and mode of hearing was still significant in the lack of a ceiling effect [*F*_2, 28_ = 24.25; *p* < 0.0001]. The result confirms that CI users are significantly more affected by interruption with silent intervals than are the NH listeners.

**TABLE 3:** Repeated measures two-way ANOVA on intelligibility scores of NHnorm and CI users with rate of interruption as within-subject and mode of hearing as between-subject factor

Source	*F* value	Significance (*p* value)
(i) 50 % duty cycle		
Interruption	*F*(3.01, 42.07) = 38.15	*p* < 0.0001
Mode	*F*(1, 14) = 231.7	*p* < 0.0001
Interruption × mode	*F*(3.01, 42.07) = 15.41	*p* < 0.0001
(ii) 75 % duty cycle		
Interruption	*F*(5, 70) = 30.11	*p* < 0.0001
Mode	*F*(1, 14) = 97.23	*p* < 0.0001
Interruption × mode	*F*(5, 70) = 25.79	*p* < 0.0001

## **EXPERIMENT 2. PERCEPTION OF INTERRUPTED SPEECH COMPARED BETWEEN CIs AND NOISE-BAND VOCODING (NHVoc)**

Speech transmitted via a CI is poor in spectro-temporal details. This poor spectro-temporal resolution may reduce bottom-up cues in effect affecting interrupted-speech perception. One way to systematically explore the effect of reduced spectro-temporal resolution on interrupted-speech perception is using a noise-band vocoder. Noise-band vocoders (Shannon et al. 1995), though not detailed emulations of the actual CI device, follow the basic principles of CI signal processing and degrade spectro-temporal information in a comparable way. Experiment 2 was run with NH listeners presented with noise-band vocoded speech to explore the effect of spectro-temporal degradation on interrupted-speech perception in CI users.

### Participants and Procedure

The NH participants from experiment 1 also participated in experiment 2. The stimuli, introduction of interruptions and procedures were the same as mentioned in experiment 1. The main difference in procedures was that the sentences were first interrupted and then noise-band vocoded with eight channels, which has been observed to produce levels of speech performance functionally similar to high performing CI users (Shannon et al. 1995; Friesen et al. 2001; Chatterjee et al. 2010; Başkent and Chatterjee 2010; Başkent 2012). Further, the training and baseline measurement were done with vocoded sentences. The NHVoc session always followed the NHnorm session on the same day.

For vocoding, the speech signal, ranging from 150 to 7 kHz in bandwidth, was filtered into eight analysis bands using a set of Butterworth bandpass filters (order 6, roll-off 36 dB/octave). The cutoff frequencies of the analysis bands were determined on the basis of Greenwood’s mapping function (Greenwood 1990) and using equal cochlear distance. The envelope was extracted for each band using full-wave rectification and a low-pass Butterworth filter (order 3, roll-off 18 dB/octave, cutoff frequency 160 Hz). If the envelope cutoff frequency is higher than F0, then the amplitude modulation of the envelope can encode periodicity and intonation information. But temporal cues to pitch are less effective as sensitivity to modulation decreases with modulation frequency above 200 Hz (Souza and Rosen 2009). Keeping this and the F0 of the speaker of the speech material (120 Hz) in view, the envelope cutoff frequency of 160 Hz was chosen. The carrier noise bands were produced by filtering white noise with the same set of bandpass filters. The extracted envelope for each channel was then used to modulate the noise carrier of that channel. The amplitude-modulated noise bands from all the channels were then combined to produce the noise-band vocoded speech. The root mean square of the resulting signal was adjusted to that of the original signal.

### Results

**FIG. 3:**
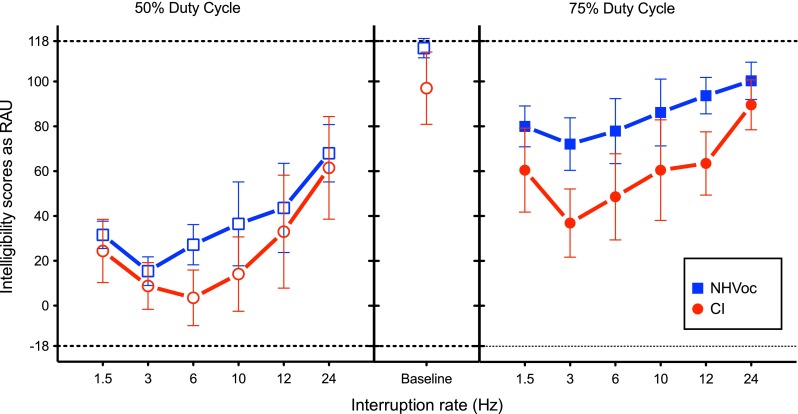
Similar to Fig. 2, except that the data for NH control group is shown for eight-channel noise-band vocoding (NHVoc; *blue square*).

Figure 3 shows the data from NH listeners tested with noise-band vocoding (NHVoc). The sentence-identification baseline for NHVoc was significantly better than the baseline for the CI users [CI and NHVoc; *t*_7.99_ = 3.02, *p*_adj_ = 0.025], but very similar to the baseline of NHnorm [NHVoc and NHnorm; *t*_7_ = 1.29, *p*_adj_ = 0.23]. The baseline data showed that 8-channel vocoder processing alone may underestimate the intelligibility deficits of uninterrupted sentences in CI users.

Although the vocoding alone did not have a significant effect on the intelligibility, the addition of interruption to the vocoded speech resulted in a significant drop in intelligibility for NHVoc for all interruption conditions [Dunnett’s, *p* < 0.0001]. Nonmonotonicity in intelligibility scores with respect to the rates of interruption was observed with NHVoc listeners as well, with the slowest 1.5-Hz rate of interruption scoring better than 3 and 6 Hz but lower than other faster rates of interruption.

To test if the signal degradation simulated with standard noise-band vocoding alone could explain CI users’ deficit in interrupted speech intelligibility, a two-way mixed model ANOVA, with interruption rate as within-subject factor and mode of hearing (NHVoc vs. CI) as between-subject factor, was performed for each duty cycle (Table 4). For both the duty cycles, the main effect of interruption rate was found to be significant, with significant difference occurring only at 6-Hz interruption rate for the 50 % duty cycle [6 Hz *p*_adj_ < 0.01; all others *p*_adj_ ≥ 0.05], but at all the interruption rates for the 75 % duty cycle [for all interruption rates, *p*_adj_ < 0.05].

**TABLE 4:** Repeated measures two-way ANOVA on intelligibility scores of NHVoc and CI users with rate of interruption as within-subject and mode of hearing as between-subject factor

Source	*F* value	Significance (*p* value)
(i) 50 % duty cycle		
Interruption	*F*(5, 70) = 38.04	*p* < 0.0001
Mode	*F*(1, 14) = 5.604	*p* < 0.05
Interruption × mode	*F*(5, 70) = 1.691	*p* = 0.15
(ii) 75 % duty cycle		
Interruption	*F*(5, 70) = 35.66	*p* < 0.0001
Mode	*F*(1, 14) = 17.79	*p* < 0.0001
Interruption × mode	*F*(5, 70) = 3.594	*p* < 0.001

The main effect of mode of hearing (CI vs. NHVoc) was also found to be significant for both the duty cycles, though the *p* values differed. The interaction of the mode and the rate of interruption was not significant for the 50 % duty cycle, but significant for the 75 % duty cycle. Figure 3 also reveals that, as the duty cycle increased, the intelligibility scores of both the groups increased, but the difference between the scores for the two modes of hearing also increased. This means that compared to the CI group, the NHVoc group was better able to utilize the extra spectro-temporal cues with longer duty cycle to improve the intelligibility of interrupted speech.

These results indicate that not only is the intelligibility of interrupted speech in CI users lower than that of NH listening to a standard eight-channel noise-band vocoded speech for both the duty cycles but also the CI users could not match the NHVoc in terms of taking advantage of longer looks with longer duty cycle and faster looks with faster interruption rates. This helps us speculate that the difference between NHnorm and CI groups in this study would be due to more factors than only the signal degradation. Two of these factors were investigated in the next experiment.

## **EXPERIMENT 3. PERCEPTION OF INTERRUPTED SPEECH COMPARED BETWEEN CIs AND NOISE-BAND VOCODING: AGE AND SENTENCE-IDENTIFICATION BASELINE MATCHED (NHVocM)**

The intelligibility of interrupted speech for CI users was found to be worse than that of the NHVoc group. Whereas there was a large variation in the age range of the CI users, many of whom were elderly, the NH listeners were comparatively younger. Also, although CI users with relatively high clinical scores were selected for this study, their average sentence-identification baseline score for the material used in this study had a large variation and was significantly lower than that of the NHnorm and NHVoc groups. The difference between the interrupted-speech perception of CI and NH groups observed in experiments 1 and 2 could be due to these reasons.

To minimize the potential confounds from older age and lower baseline speech intelligibility scores of CI users, an age-matched NH listener (NHVocM) was used for each individual CI participant and was presented with the noise-band vocoded speech in a configuration which resulted in matching his/her sentence-identification baseline score to that of the corresponding CI user.

### Participants and Procedure

Eight NH listeners (three males and five females; 28 to 75 years; average age 53.8) who were age matched (±2 years) with individual CI users participated in this experiment. These participants were different from the NH participants of experiments 1 and 2.

A pilot study was run before the main experiment for each NHVocM participant to determine the suitable combination of number of channels (six or eight channels) and filter order (1, 2, 3) for the main experiment. For the pilot, along with the sentences, phoneme-identification scores using NVA corpus was also used. Processed with a combination of six or eight channels and 1st, 2nd or 3rd filter order, six lists of the sentence corpus and six lists from the word corpus were presented in the pilot session.

**FIG. 4:**
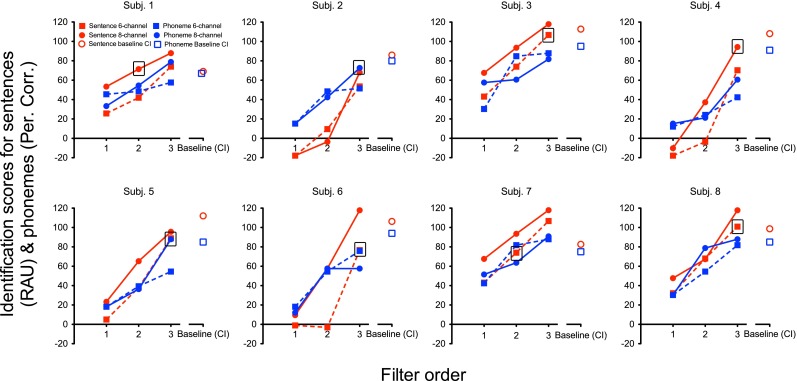
Scores used for baseline matching for experiment 3. Individual panels show the identification scores for sentences (RAU; *red open circle*) and phonemes (percent correct; *blue open square*) of the target CI user to be matched to an individual age-matched NH participant. The corresponding sentence (*colour red*) and phoneme (*colour blue*) scores for the NHVocM participant plotted for eight-channel (*solid line with square*) and six-channel (*dotted line with circle*) noise-band vocoding, shown as a function of the three filter orders. The channel-filter order combination selected for main experiment is marked with a *black square*.

That channel and filter order combination was chosen for the main experiment which provided the scores matching suitably with the sentence-identification baseline of the CI users. The data from baseline-matching pilot is shown in Figure 4.

The main experiment was conducted the same way as experiment 1, but with noise-band vocoding with the combination of channel and filter order as obtained from the pilot.

### Results

**FIG. 5:**
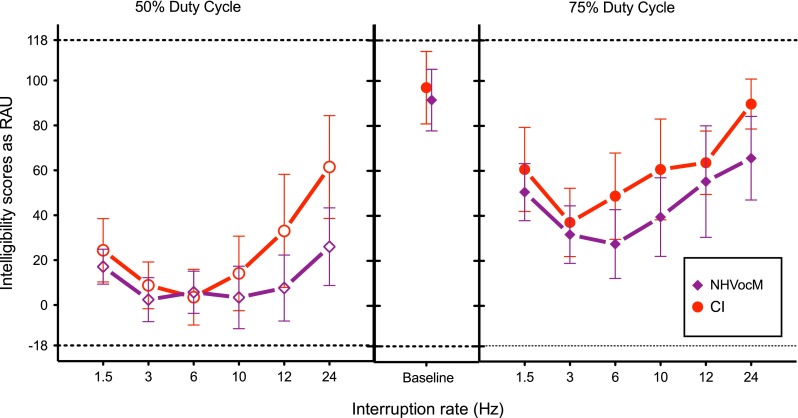
Similar to Fig. 2, except that the data for the NH control group is shown for matched noise-band vocoding (NHVocM; *purple diamond*).

Figure 5 shows the data from NHVocM and CI groups. The data showed that the sentence-identification baseline of NHVocM matched well with the CI users’ baseline [*t*_13.65_ = −0.74, *p*_adj_ = 0.47]. The performance with all of the interruption conditions was significantly different than the sentence-identification baseline intelligibility [Dunnett’s, *p* < 0.0001], indicating that just like the CI users, the NHVocM group suffered a loss of intelligibility due to interruption with silent intervals. This loss was mitigated only incompletely with longer duty cycle or faster interruption rates. Similar nonmonotonicity of intelligibility scores with respect to the rates of interruption was observed with NHVocM listeners as with other groups of listeners. The intelligibility scores with the slowest rate of interruption were higher than medium rates of interruption, but lower than the fastest rate of interruption.

Two-way ANOVAs with the rate of interruption as the within-subject factor and the mode of hearing as the between-subject factor between NHVocM and CI scores indicated a significant effect of interruption for both the duty cycles (Table 5). A post hoc test on each duty cycle showed that the CI and the NHVocM scores significantly differed only for the 50 % duty cycle at 24-Hz interruption rate [50 % duty cycle, 24 Hz, *p*_adj_ = 0.023, all others *p*_adj_ > 0.05; 75 % duty cycle, for all *p*_adj_ > 0.05]. Thus, the difference between these two groups was driven by only the fastest rate of interruption at the 50 % duty cycle.

**TABLE 5:** Repeated measures two-way ANOVA on intelligibility scores of age and sentence-identification baseline matched NH listeners (NHVocM) and CI users with rate of interruption as within-subject and mode of hearing as between-subject factor

Source	*F* value	Significance (*p* value)
(i) 50 % duty cycle		
Interruption	*F*(5, 70) = 28.26	*p* < 0.0001
Mode	*F*(1, 14) = 5.726	*p* < 0.05
Interruption × mode	*F*(5, 70) = 6.264	*p* < 0.0001
(ii) 75 % duty cycle		
Interruption	*F*(5, 70) = 35.63	*p* < 0.0001
Mode	*F*(1, 14) = 4.369	*p* = 0.055
Interruption × mode	*F*(5, 70) = 2.315	*p* = 0.053

The mode of hearing was significant for the 50 % duty cycle but not for the 75 % duty cycle. The rate of interruption and the mode of hearing interacted significantly for the 50 % duty cycle but not for the 75 % duty cycle.

Thus, the results show that when the NH listeners, who are age matched to the CI users, are presented with baseline-matching noise-band vocoding, their intelligibility of the interrupted speech and the benefit of increased duty cycle is found to be similar to that of the actual CI users. This underscores the importance of the factors of aging and lower intelligibility of uninterrupted speech in governing the performance of CI users with speech interrupted with silent intervals. This indicates that top-down cognitive factors associated with aging can have an effect on integration of speech glimpses. It also indicates that not matching the age and baseline performance in studies with CI users and NH listeners may lead to underestimating the performance of CI users.

## **DISCUSSION**

This study was conducted to systematically test the effect of silent intervals on speech intelligibility in CI users. The quality, frequency and duration of the glimpses were expected to affect the integration and restoration of the interrupted speech. To test the effect of the spectro-temporal quality of glimpses, the performance of CI users was compared with the performance of NH listeners, tested with and without degradations of noise-band vocoding. The number of glimpses per second was manipulated systematically with interruption rate, and the duration of the glimpses was manipulated with duty cycle. The perception of speech interrupted with silent intervals was expected to be poor for the CI users, which may be related to the difficulty experienced by CI users in understanding speech in noise.

The collective effect of the loss of fine spectro-temporal cues as represented by the standard eight-channel noise-band vocoder and wider range of age was explored by comparing the results of the NH listeners tested with noise-band vocoding with those of the actual CI users. Finally, out of these additional factors, the combined effect of age and intelligibility of uninterrupted speech was explored by comparing the results of age-matched NH listeners tested with specific noise-band vocoding parameters that provided matching baseline performance.

### Interrupted-Speech Perception in CI Users

The experiment found that the introduction of silent intervals to speech leads to a significant loss of intelligibility for the CI users (Nelson and Jin 2004; Chatterjee et al. 2010; Başkent and Chatterjee 2010). But unlike the findings reported by Nelson and Jin (2004), the speech perception did not stay at floor level for all interruption rates at the 50 % duty cycle which was a common parameter for both studies. Furthermore, significant intelligibility was found for all interruption rates for the 75 % duty cycle for CI users, indicating not only the ability of CI users to perceive interrupted speech but also the importance of using large number of conditions for studies with CI users.

Although the details of the CI participants of Nelson and Jin (2004) are not available, the disparity between the performance of CI users in the present study and the Nelson and Jin study could be because all of the CI participants in the present study were ‘star-subjects’ with very high open-set monosyllabic word recognition scores. Apart from this, several of the CI participants in the present study also had residual hearing which could have resulted in high intelligibility scores for them. Residual hearing may help in better speech intelligibility in CI users due to better transmission of voice pitch cues and other information from better resolved low frequencies. The difference in speech material used in the two studies may have also contributed to the observed differences in the two studies.

The degree and the nature of the loss of intelligibility due to silent intervals in the CI users were found to be different from those in the NH listeners. In agreement with the previous studies (Miller and Licklider 1950; Powers and Speaks 1973; Nelson and Jin 2004; Shafiro et al. 2011; Bhargava and Başkent 2012), the NH listeners suffered substantial loss of intelligibility at few interruption and duty cycle conditions. In comparison, the CI users suffered the loss in almost all interruption and duty cycle conditions.

The reason for the difference in interrupted-speech perception among CI users and NH listeners could be that full-spectrum speech is rich with acoustic cues, e.g. periodicity cues for voice pitch, temporal fine structure cues for formant patterns and place of articulation, and envelope cues for manner of articulation (Rosen 1992; Assmann and Summerfield 2004). Despite the addition of silent intervals, many of these acoustic cues remain available in the intact portions of the normal speech helping the NH listeners to predict and restore the interrupted speech. As compared with the full-spectrum speech, CI-processed speech contains less information as it is devoid of fine spectro-temporal details and contains only temporal envelopes from few spectral bands (Loizou 1998). These envelope cues provide important information about the changes in syllabic (e.g. word onset and offset, speaking rate, prosody, etc.) and phonetic (voicing, manner, etc.) segment constituents (Shannon et al. 1995; Assmann and Summerfield 2004; Fogerty and Humes 2012) which is crucial for understanding speech for CI users (Rosen 1992; Tasell et al. 1992; Fu et al. 2004; Nie et al. 2006). Addition of silent intervals disrupts these crucial envelope cues, and in the absence of spectral details and fast fluctuating temporal information, CI users are not able to predict and restore the missing speech portions. This leads to a loss of intelligibility of interrupted speech in CI users. Thus, the primary finding of this study is that though CI users are able to integrate the glimpses of speech interrupted with silence and are capable of taking advantage of extra auditory cues availed by longer intact samples, the extent of this is significantly lesser than in NH listeners. This means that the interrupted-speech perception in CI users in this study is deficient as compared to the NH listeners. The general trend of the results is similar to other studies who reported that CI users could integrate the interrupted speech information, though not as well as the NH listeners (Gnansia et al. 2010). They also speculate that the deficit stems at least partly from poor representation of temporal fine structure and fine spectral details.

In the first experiment comparing the performance of NH listeners and CI users, the intelligibility of interrupted speech decreased for the medium rates (3–6 Hz) as compared with the slowest rate of interruption. Then, the intelligibility increased for both NH listeners and CI users as the rate of interruption increased to 10 Hz and above. We tested modulation masking as an explanation for this nonmonotonicity. The envelope modulations of the range 2–16 Hz are most important for speech intelligibility (Houtgast and Steeneken 1985; Drullman et al. 1994; Füllgrabe et al. 2009) as these are also the rates of modulation for word, syllabic and phonemic segments (Plomp 1984; Assmann and Summerfield 2004). Modulation of envelope with silent intervals at rates in this range is likely to interfere with the perception of speech sounds at the word, syllabic and phonemic levels, leading to a loss of intelligibility. Since NH listeners do not rely on envelope cues as strongly as CI users, they are less likely to be affected. But an inspection of modulation spectra from uninterrupted and interrupted vocoded sentences showed that modulation masking (at least as depicted in modulation magnitude spectra) does not independently predict the results of our study. This may be because speech intelligibility does not rely on modulation magnitude alone. It is a complex phenomenon that involves several other factors like coarticulation, stress, timing and contextual cues whose role cannot be accounted for by only modulation magnitude.

A plausible reason for the nonmonotonicity is the amount of useful context of speech stimulus at various rates of interruption. At the slowest rate of interruption, there are longer portions of intact speech, with the possibility of entire words escaping obliteration, providing enough linguistic and auditory cues to make speech intelligible. The medium rates of interruption in the present study match the syllabic rates of speech (Verhoeven et al. 2004; Edwards and Chang 2013), which leads to the possibility that the silent intervals obliterated syllables, causing intelligibility to be lower than the slowest rate of interruption. At fast rates of interruption, the listeners can access multiple ‘looks per word’ or even per syllable (Miller and Licklider 1950), which presumably helps filling in for the missing parts (Wang and Humes 2010). But there was a significant difference among the two groups, as the advantage of the faster rates of interruption was not as large for CI users as it was for NH listeners.

The longer duty cycle was expected to produce higher intelligibility. A reason for this is that, with longer duty cycle, longer intact samples are available which are likely to contain more intact acoustic cues leading to better intelligibility (Bhargava et al. 2014). For example, longer samples have a better chance of conveying the time-varying F0 cues of the sentence which facilitate the integration of speech glimpses during interrupted contexts (Darwin et al. 2003). Another reason could be that with shorter duty cycle, the speech segments separated by long duration of silent intervals were likely to be processed as isolated fragments, disrupting the integration of speech segments and causing a loss of speech intelligibility (Huggins 1975). With longer duty cycle, the gaps could be easily bridged and the speech segments could be combined more readily, improving intelligibility. In case of NH listeners, longer duty cycle contained more envelope cues, as well as temporal and spectral fine structure cues. For the CI users, on the other hand, longer duty cycle mostly provided more intact envelope cues. Thus, though both NH listeners and CI users could obtain advantage from longer duty cycle as compared with shorter duty cycle, NH listeners could obtain greater advantage than CI users.

### Role of Limited Spectral and Temporal Envelope Cues

To test if the disruption in envelope cues combined with limited spectro-temporal resolution was indeed the reason behind CI users’ low interrupted speech intelligibility, young NH listeners were tested for interrupted-speech perception with 8-channel noise-band vocoding (NHVoc). By itself, the 8-channel noise-band vocoding did not lead to a significant loss of intelligibility, as the sentence-identification baseline for NHVoc was significantly higher than the sentence-identification baseline for CI and in fact as good as the sentence-identification baseline for NHnorm (Figs 2 and 3). The interrupted-speech perception as well as the advantage of longer duty cycle also stayed better for NHVoc group than for CI users. This leads to the conclusion that the loss of spectro-temporal resolution as simulated by 8-channel noise-band vocoding alone seems to have an effect on interrupted-speech perception, but it does not capture the loss of interrupted-speech intelligibility experienced by the CI users. Additional factors need to be examined in order to account for the difference between the performances of the two groups.

### Role of Additional Factors

There are several factors affecting intelligibility of speech for a CI user that the typical noise-band vocoding does not account for, e.g. attack and release times of automatic gain control (Khing et al. 2013), channel interactions (Laneau et al. 2006), the amount and location of healthy neurons in the cochlea (Bierer 2010), insertion depth of the electrode array (Başkent and Shannon 2005), residual hearing (Turner et al. 2004; Başkent and Chatterjee 2010), etc. Limitations induced by such factors could have contributed to the lower baseline intelligibility for the CI users as compared to the NHVoc listeners. Baseline intelligibility is a measurement of a listener’s use of bottom-up cues when no other external degradations are present. Since interrupted-speech perception involves employing bottom-up cues from the intact glimpses to restore the missing information, factors leading to low baseline intelligibility may also lead to low interrupted-speech perception. Thus, low interrupted-speech perception of CI users as compared to NHVoc listeners may be a projection of factors specific to the CI group, also causing low baseline scores.

Apart from the baseline intelligibility, the average ages of the participants in the two groups were also different, with CI listeners having a wider range of ages than the NH participants. Some of the CI users of the present study were considerably older than the remaining CI and NH participants. Although aging is known to affect both peripheral and cognitive mechanisms, because the NHVoc listeners were presented with vocoded speech, age differences are expected to be reflected mostly at the level of cognitive mechanisms. For example, aging is accompanied by a general slowing down in cognitive processes (Birren et al. 1980; Salthouse 1996; Wingfield 1996; Gazzaley et al. 2005). This may have influenced the effective use of top-down mechanisms in the elderly CI users. In a similar study, for example, Saija et al. (2014) have shown that the intelligibility of interrupted sentences is significantly worse in elderly NH listeners than in younger NH listeners. In fact, aging effects have been found to be evident even in middle-aged NH listeners in conditions involving masking by music, competing talker and noise (Başkent et al. 2014). Thus, the difference in interrupted-speech perception between CI and the NHVoc group could have been due to other factors related to actual CI users apart from loss of spectro-temporal resolution.

To see the combined effect of other factors associated with the actual CI users, the age difference between the NH and the CI group was mitigated by recruiting NH listeners (NHVocM) with the same age as CI users. Difference in sentence-identification baseline between the two groups was mitigated by modifying the spectral resolution of the bottom-up cues. This was achieved by noise-band vocoding the speech with vocoder configuration that resulted in similar baseline scores as those of CI users. On matching the age and baseline, it was found that the intelligibility of interrupted speech fell considerably for the vocoded speech listeners. Although the CI users scored marginally better than the vocoded speech listeners, silent interruptions affected the two groups similarly. These groups also took similar advantage of increase in duty cycle. This confirms that factors, in addition to spectro-temporal resolution, e.g. the effects of aging and the various factors governing the use of bottom-up auditory cues, may have significant influence on the interrupted-speech perception.

Our results are in agreement with predictions derived from the studies modelling the relationship between recognition scores of elements and wholes (Boothroyd and Nittrouer 1988; Boothroyd et al. 1996). The models state that the probability of recognizing the whole has a power relationship with the probability of recognizing the constituents, such that a decrease in probability of recognizing the whole would result in a larger decrease in recognizing probability of the constituents. This model predicts that a decrease in baseline would result in a larger decrease in interrupted-speech perception, which is what we observed too.

## **CONCLUSION**

To summarize, it was found that though CI users could understand interrupted speech, their interrupted-speech intelligibility was significantly worse than that of NH listeners. Aging effects, and the quality of bottom-up auditory cues, as reflected by the sentence-identification baseline intelligibility, were found to affect interrupted-speech perception performance. In CI users, reduced bottom-up auditory cues like weaker temporal pitch cues and reduced spectral cues affect the redundancy in speech signal (McKay and Carlyon 1999; Green et al. 2002), which, when combined with disruption in envelope cues due to interruptions, would impede integration and restoration. In case of older CI listeners, the age-related deficits could additionally affect the integration and restoration. This would consequently result in poorer interrupted-speech perception in CI users.

### Implications and Limitations of the Study

In noisy scenarios, as the noise masks the portions of speech, the listener has to rely on unmasked glimpses of speech to integrate and restore the masked portions of speech. The results from our interrupted-speech study indicate that age-related factors coupled with poor transmission of auditory cues from the intact speech portions may affect the restoration and integration of glimpses. Thus, the factors affecting interrupted-speech perception help to also at least partially explain the problem experienced by CI users in understanding speech in noisy environments, especially when fluctuating maskers interrupt the target speech signal (Nelson et al. 2003; Qin and Oxenham 2003; Stickney et al. 2004).

An important implication of the study is that the age and baseline intelligibility scores of CI users may have significant effect on their performance with further degradation. In studies involving both CI users and noise-band vocoded speech listeners, a mismatch in their age and baseline intelligibility scores can have significant effect on the results. Similarly, in studies simulating the performance of CI users using young NH listeners with noise-band vocoding, rendering baseline intelligibility scores at ceiling may underestimate the breakdown of speech intelligibility experienced by actual CI users.

Although important, the findings of this study must be interpreted in the light of some observations. Firstly, it should be noted that interrupted-speech perception is a simplified model of speech perception in a difficult listening scenario. Our results are based on only one type of temporal degradation, and a different type of listening scenario (e.g. with competing talkers, jittering, reverberation, etc.) may produce different results.

Secondly, although age and baseline matching seem to predict almost all the deficit in the interrupted-speech perception of CI users as compared with the noise-band vocoded speech listeners, the matching itself may not have captured all the factors responsible for the deficit. For example, despite matching the age, the differences in cognitive mechanisms associated with aging were not explicitly measured and matched. Similarly, matching of the baseline through noise-band vocoding only provided a functional match of performance between CI users and vocoded speech listeners. There are various aspects of actual implant processing that our noise-band vocoding has not taken into account, e.g. the degree of spectral spread, the effect of various aspects of transmission of modulations through electrical stimulation, effect of place-frequency mismatch, etc. See Shamma and Lorenzi (2013) for further discussion on limitation of vocoders.

Further, the speech stimuli used in the present study was spoken by only one male speaker. Comparison across studies must be done remembering that the gender of the talker may influence speech perception; e.g. Loizou et al. (1998) reported that vowels produced by male talkers were easier to identify than female talkers. Lastly, the interpretations in this study are based on performance of eight CI users who were mostly ‘star’ subjects. The low number of participants and their better performance on average warrant due caution in generalizing the results from this study on the general CI population.
